# The diabesity epidemic in the light of evolution: insights from the capacity–load model

**DOI:** 10.1007/s00125-019-4944-8

**Published:** 2019-08-27

**Authors:** Jonathan C. K. Wells

**Affiliations:** Childhood Nutrition Research Centre, UCL Great Ormond Street Institute of Child Health, 30 Guilford Street, London, WC1N 1EH UK

**Keywords:** Body composition, Capacity–load model, Diabetes, Evolution, Life course development, Public health, Review

## Abstract

The global nutrition transition, which embraces major changes in how food is produced, distributed and consumed, is associated with rapid increases in the prevalence of obesity, but the implications for diabetes differ between populations. A simple conceptual model treats diabetes risk as the function of two interacting traits: ‘metabolic capacity,’ which promotes glucose homeostasis, and ‘metabolic load’, which challenges glucose homoeostasis. Population variability in diabetes prevalence is consistent with this conceptual model, indicating that the effect of obesity varies by ethnicity. Evolutionary life history theory can help explain why variability in metabolic capacity and metabolic load emerges. At the species level (hominin evolution), across human populations and within individual life courses, phenotypic variability emerges under selective pressure to maximise reproductive fitness rather than metabolic health. Those exposed to adverse environments may express or develop several metabolic traits that are individually beneficial for reproductive fitness, but which cumulatively increase diabetes risk. Public health interventions can help promote metabolic capacity, but there are limits to the benefits that can emerge within a single generation. This means that efforts to curb metabolic load (obesity, unhealthy lifestyles) must remain at the forefront of diabetes prevention. Such efforts should go beyond individuals and target the broader food system and socioeconomic factors, in order to maximise their efficacy.

**Electronic supplementary material:**

The online version of this article (10.1007/s00125-019-4944-8) contains a slideset of the figures for download, which is available to authorised users.

## Introduction

The rapidly emerging global epidemics of obesity and diabetes (a linkage termed ‘diabesity’) are closely associated with economic development and ‘nutrition transition’ [1], a term referring to changes in human diets that have occurred over recent decades, associated with changes in how food is produced, distributed and consumed. Nutrition transition first occurred in high income countries but is now occurring fastest in low- and middle-income countries, and is thereby exposing all human populations, at different rates, to multiple factors that impact metabolism and cardiometabolic physiology [2]. Many components of nutrition transition are now conceptualised as the ‘obesogenic niche’, embracing unhealthy diets, sedentary behaviour, disturbed sleep and many related pressures emanating from the broader environment. While proximate causes of obesity and metabolic dysfunction are well-established, an evolutionary perspective helps our understanding of *why* humans are prone to diabetes in obesogenic environments.

Susceptibility to diabetes is not exclusive to humans, and is also observed in primates kept in captivity [3]. Research on rodents has highlighted many relevant components of mammalian physiology and behaviour [4]. Nevertheless, we need to understand both why humans as a species are currently prone to diabesity, and why some populations and individuals are more susceptible than others. This review aims to address these questions.

### The basic metabolic model

Obesity represents a state of excess body fatness, usually assessed relatively crudely using BMI. Diabetes encompasses a constellation of conditions in which blood sugar regulation is impaired [5], which can lead to tissue damage and elevated cardiovascular risk, but my focus here is on two types closely associated with obesity: type 2 diabetes mellitus and gestational diabetes mellitus (GDM), which propagates the effects of maternal metabolic dysfunction to fetal development. Type 2 diabetes was initially considered an ‘adult-onset’ disease, with obesity the primary phenotypic risk factor. It is now characterised as a ‘two-hit’ disease, where the effects of insulin resistance in muscle tissue are exacerbated by the inability of pancreatic beta cells to supply adequate insulin to compensate [6].

In the 1990s, classic epidemiological studies showed that fetal and infant undernutrition reduce growth of the pancreas and muscle mass, interpreted as developmental adjustments that protect the vulnerable brain at the cost of increased diabetes risk in later life (the thrifty phenotype hypothesis) [7, 8]. This reconceptualised type 2 diabetes as a condition in which adult obesity and unhealthy lifestyle promote insulin resistance, while early growth constraint contributes to pancreatic beta cell dysfunction.

**Fig. 1:**
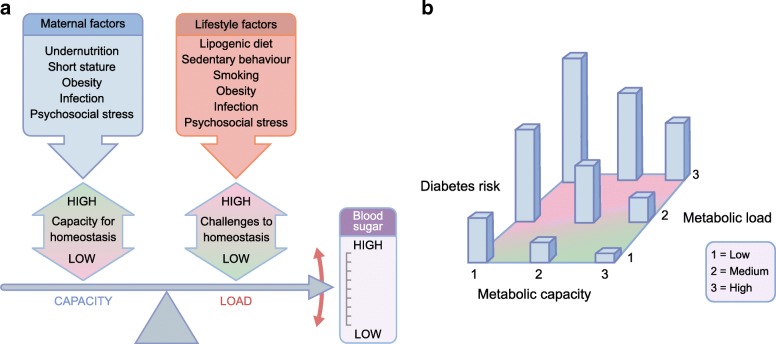
The capacity–load conceptual model. (**a**) Greater metabolic capacity promotes glycaemic homeostasis; however, the development of metabolic capacity in early life is adversely influenced by several components of maternal malnutrition. Metabolic load is detrimental for glycaemic homeostasis, leading to higher blood sugar levels, and a number of components of an individual’s lifestyle exacerbate this effect. (**b**) Three-dimensional diagram of the interactive associations of metabolic capacity and load with diabetes risk. (**a**) Adapted from [30] under the terms of the Creative Commons Attribution License (CC BY), which permits use, distribution or reproduction in other forums; (**b**) adapted with permission from [9], ©2010 Wiley-Liss, Inc. This figure is available as part of a downloadable slideset

Building on the thrifty phenotype hypothesis, I developed a broader model of disease risk, emphasising two generic risk factors: (1) metabolic capacity, incorporating traits that promote the capacity for homeostasis, and (2) metabolic load, incorporating phenotypic traits that challenge homeostasis [9, 10] (Fig. 1a). Key aspects of metabolic capacity in relation to diabetes are pancreatic function (insulin production) and muscle mass (key to glucose clearance), both of which are strongly influenced by fetal and infant growth [9, 10]. Key components of load relating to diabetes are adiposity (especially abdominal adiposity), dietary glycaemic load, sedentary lifestyle and psychosocial stress, all of which perturb glycaemic control and promote oxidative stress and chronic inflammation [10], though other components of load, such as infection and smoking, also have an impact on cardiometabolic function. In this sense, metabolic load can be considered part of the individual’s ‘extended phenotype’ [11], integrating physical traits with both voluntary behaviours and involuntary environmental exposures.

Diabetes risk is expected to increase directly with load and inversely with capacity, through interactive dose–response associations (Fig. 1b), and this prediction is closely matched by data from large cohorts: birthweight (a useful marker of capacity) shows only a weak association with diabetes among adults living healthy lifestyles (low load), but a strong inverse association with diabetes risk among adults with multiple components of unhealthy lifestyle (high load) [12]. Notably, diabetes risk increases with age as beta cell function deteriorates [13], as discussed further below in the section on ‘Evolutionary approaches to contemporary variability in diabetes risk’.

The thrifty phenotype hypothesis identified the combination of early growth variability and later overnutrition as key to the life course aetiology of diabetes. A complementary mechanism comprises genetic factors (e.g. the glucokinase gene) that contribute to both low birthweight and later diabetes risk (the thrifty genotype hypothesis) [14]. Such genetic reductions in metabolic capacity are discussed further below; however, the validity of the thrifty phenotype hypothesis is supported by the increased diabetes risk in offspring conceived after, vs before, the mother herself developed diabetes [15].

As maternal obesity became more common, a second developmental pathway emerged: maternal obesity and GDM expose the fetus to excess fuel supply, inducing changes to pancreatic phenotype along with excess fat deposition before birth [16, 17]. In this scenario, the mother once again constrains the metabolic capacity of the offspring, through her inability to maintain glycaemic homeostasis during pregnancy, raising the metabolic load of the offspring before birth with adverse long-term metabolic consequences. The association of birthweight with diabetes is therefore J-shaped, with increased type 2 diabetes risk at low and high birthweights [18].

This basic physiological model allows us to consider the implications of phenotypic variability for diabetes risk across different timescales. Over the very long term, for example, hominins and humans appear to have evolved both lower levels of muscle mass and higher adiposity relative to other primates, especially in females [19, 20]. However, the ‘toxic’ effects of higher fatness in human females have been partially resolved by the co-evolution of a more gluteo-femoral distribution of fat, which appears beneficial for insulin sensitivity [21, 22].

Overall, this suggests that humans evolved an elevated susceptibility to diabetes compared with other primates, which may be activated on exposure to obesogenic environments [20]. Consistent with this hypothesis, markers of metabolic capacity and load also correlate with variability in diabetes prevalence across contemporary human populations.

### Explaining worldwide population variability in diabetes prevalence

To test the capacity–load model across populations, a database was compiled for both risk markers. Country-specific data on obesity and diabetes prevalence for 2014 were downloaded from www.ncdrisc.org. The majority of neonates in low- and middle-income countries are not yet weighed; hence, mean birthweight could only be obtained for 80 countries, using studies conducted before 1990 [23]. Female adult height, a marker of the maternal capacity to promote fetal growth in the next generation, was obtained for 1996 from the NCD Risk Factor Collaboration [24]. These data, collected across 80 countries of varying levels of economic development, allow several important concepts to be illustrated.

First, the data support the overall capacity–load model (Table 1). For both sexes, regression models demonstrate negative associations of diabetes prevalence with birthweight and female adult height, and positive associations with adult obesity prevalence. These findings support cohort studies [12] confirming adult obesity and birthweight as key markers of metabolic load and capacity, respectively (though capacity may continue to develop in postnatal life). The models also identify female adult height as an independent index of maternal metabolic capacity, relevant to promoting growth of the next generation. The models explain substantially more variance in diabetes prevalence for women than men (~70% vs ~34%), which might relate to sex differences in the relationship between BMI and body composition.

**Table 1:** Regression models of diabetes prevalence on markers of metabolic capacity and load, by sex

Predictor	β-coefficient	SE	*t* value	*p* value	*r* ^2^
Women (*n* = 80 countries)					
Constant	0.760	0.110	6.89	<0.001	0.699
Birthweight (kg)	−0.062	0.020	−3.03	0.003	
Female height (cm)	−0.004	0.001	−3.09	<0.001	
Female obesity prevalence (%)	0.362	0.028	12.93	<0.001	
Men (*n* = 80 countries)					
Constant	0.811	0.144	5.64	<0.001	0.340
Birthweight (kg)	−0.044	0.024	−1.81	0.073	
Female height (cm)	−0.004	0.001	−3.69	<0.001	
Male obesity prevalence (%)	0.297	0.048	6.25	<0.001	

SE, standard error of the β-coefficient

Data sources:

Obesity and diabetes prevalence (2014): www.ncdrisc.org

Birthweight (studies conducted before 1990): [23]

Female adult height (1996): [24]

**Fig. 2:**
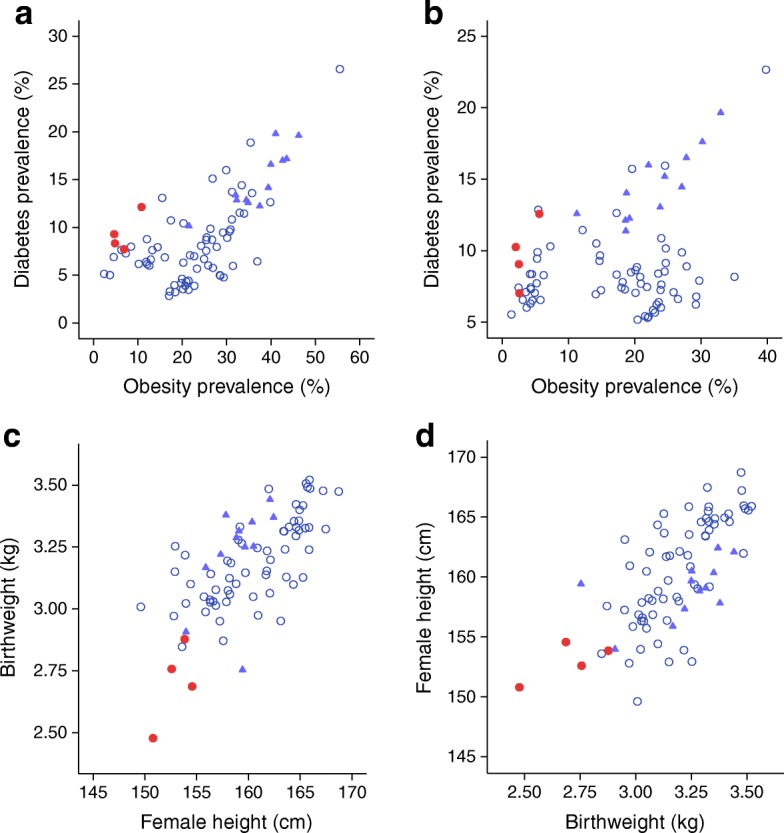
Patterns of diabetes prevalence and metabolic risk markers across 80 countries. (**a**) Diabetes prevalence and obesity prevalence in women. (**b**) Diabetes prevalence and obesity prevalence in men. (**c**) Birthweight and adult female height. (**d**) Adult female height and birthweight. Red circles, South Asian population; blue triangles, Middle Eastern and North African populations; white circles, all other populations. Data sources: Obesity and diabetes prevalence (2014): www.ncdrisc.org; Birthweight (studies conducted before 1990): [23]; Female adult height (1996): [24]. This figure is available as part of a downloadable slideset

Second, plots of the data indicate that every population is affected by type 2 diabetes to some degree, and using this approach allows high-risk populations to be evaluated, of which two examples are discussed here. South Asian populations have relatively high diabetes prevalence, given their obesity prevalence (Fig. 2a, b). According to the conventional BMI cut-off of 30 kg/m^2^, obesity prevalence remains very low in South Asia [25]. However, these populations have lower levels of lean mass for their height, thus reducing their overall BMI [26]. Using a more appropriate cut-off of 27 kg/m^2^ [27], or waist circumference cut-offs, the prevalence of obesity is substantially higher and may approach 50% in some urban populations [28, 29]. The populations of South Asian countries show both low birthweight and low adult height, but even more importantly, birthweight is low after taking female adult height into account (Fig. 2c). These patterns indicate that the range of metabolic capacity is generically very low in South Asian populations, and helps understand why diabetes typically develops at relatively low BMI thresholds [30].

Another high-risk group comprises countries from North Africa and the Middle East [31]. These countries cluster at the higher end of the range for both obesity and diabetes prevalence in each sex, highlighting the costs of high adult metabolic load (Fig. 2a, b). Paradoxically, when plotted against adult female height, birthweight appears relatively high in these countries (Fig. 2c), with the exception of Kuwait, where the data appear to be major outliers. However, these birthweights may not indicate high metabolic capacity, and previous evolutionary analyses have suggested that high rates of consanguineous marriage contribute to lower birthweights in these populations [32]. Given that the obesity epidemic emerged early in this global region [33], high birthweights relative to female height suggest elevated neonatal adiposity, consistent with reports of high levels of GDM and macrosomic infants in these countries [34, 35]. Indeed, the reverse plot shows that these populations have relatively low maternal height for their birthweight (Fig. 2d), suggesting that lean mass at birth is already relatively low and constrains linear growth. These patterns require direct confirmation, but I suggest that the high prevalence of diabetes in North African and Middle Eastern populations may arise through the combination of relatively high metabolic load in adult life, and both low metabolic capacity and high metabolic load at birth.

Overall, the high diabetes prevalence in each of these high-risk groups supports the two overarching pathways to elevated diabetes risk highlighted earlier, namely, low metabolic capacity associated with low birthweight (South Asia), or the early emergence of high metabolic load associated with high birthweight (North Africa and the Middle East). In each case, the elevated susceptibility to diabetes is subsequently triggered by high metabolic load in later life. This approach can be applied to other high-risk groups, such as Australian aboriginal populations and Pacific Islanders [10].

### Evolutionary approaches to contemporary variability in diabetes risk

To develop an evolutionary perspective on the diabesity epidemic, we need a broad theoretical model of how ecological stresses shape variability in both metabolic capacity and load, whether through long-term genetic adaptation or through life course plasticity.

**Fig. 3:**
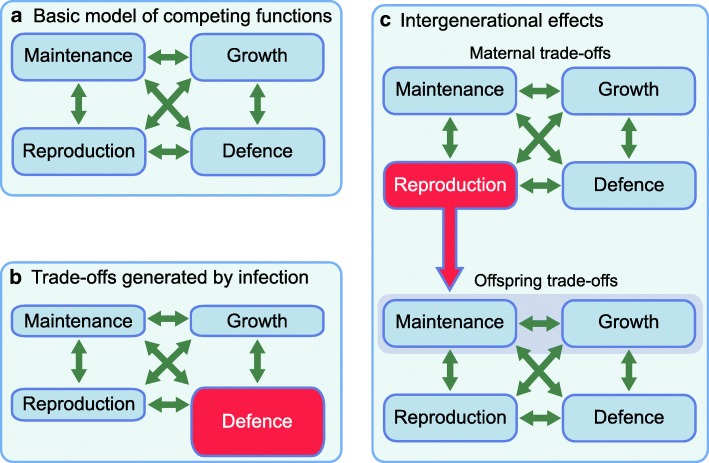
Life history theory and trade-offs in energy allocation. (**a**) The basic model assumes that energy must be allocated between four competing traits. (**b**) An example of a trade-off, where activating immune function to overcome an infection temporarily reduces energy availability for other functions. (**c**) An intergenerational model, where the energy budget of offspring during early life is determined by the life history trade-offs made by the mother. Offspring allocations to maintenance and growth (highlighted in grey) are especially sensitive to this maternal allocation during early ‘critical windows of development’, generating life-long effects on diabetes risk, as summarised in Fig. 5. This figure is available as part of a downloadable slideset

Evolutionary life history theory assumes that all organisms are under selective pressure to harvest resources from the environment, and to allocate them to biological functions to maximise fitness [36]. Those organisms making the best use of energy over the lifespan should receive the highest fitness pay-offs. Energy is allocated between four functions, namely, maintenance (effectively, homeostasis), growth, reproduction and defence against pathogens and predators [36–38]. Increased investment in any one trait reduces energy allocation to the other traits, resulting in trade-offs between them [36]. Similarly, factors affecting maternal life history trade-offs affect the allocation of energy to the next generation (Fig. 3).

Life history theory links readily with the capacity–load model described above [10]. First, early investment in metabolic capacity benefits homeostatic ‘maintenance’ throughout the life course. Second, many trade-offs that promote immediate survival and reproduction also elevate metabolic load. Examples include the stress response, immune activation and energy storage in adipose tissue, since this promotes chronic inflammation. On this basis, ecological stresses that impact metabolism inherently shape diabetes risk in a cumulative manner throughout the life course. The same approach helps explain the association of diabetes risk with age. The disposable soma theory assumes that fitness is maximised by investing in homeostasis in proportion to life expectancy, as additional investment produces no fitness returns. Shorter life expectancy therefore predicts earlier deterioration of homeostasis, helping explain why poor fetal growth is associated with both all cause premature mortality [39] and elevated diabetes risk [7]. Using this approach, we can now consider both long-term and life course variability in metabolic traits.

### Long-term trends in capacity and load

What could have driven changes in birthweight and height, the markers of metabolic capacity, over evolutionary timescales? At a proximate level, the most obvious factor is a low nutritional supply to the fetus and infant, propagating effects to adult height. However, such nutritional stresses might not necessarily originate during fetal life in each generation, but, rather, accumulate across the life course and thereby impact the maternal capacity to nourish the fetus.

Figure 2c shows a strong correlation across populations between maternal height and birthweight (*r* = 0.70, *p* < 0.001). Accordingly, long-term trends in stature are expected to drive complementary birthweight trends [40]. Data from the archaeological record indicate that 10,000 years ago, for example, inhabitants of the Indian subcontinent were 15–20 cm taller than today [41], although they also appear to have maintained a thin physique throughout this period. The 10,000 year decline in height predicts a 20% decline in birthweight from 3.44 kg to 2.80 kg [30]. Several proximate mechanisms may have driven this recent height decline.

**Fig. 4:**
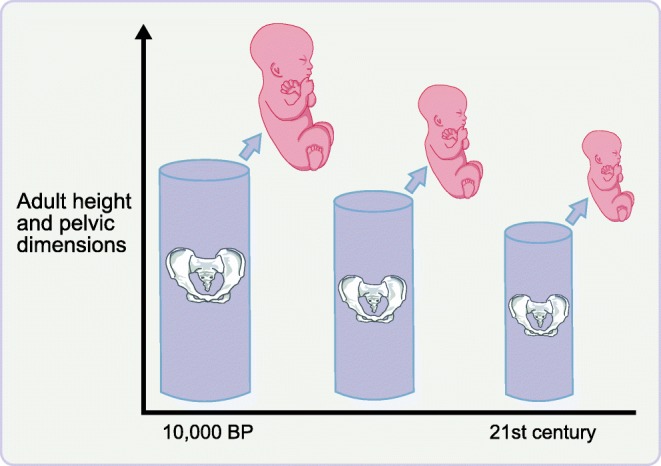
Schematic diagram illustrating how declines in adult height across generations drive a reduction in pelvic dimensions, which, in turn, forces a reduction in birthweight. This biological mechanism may have played a key role in the emergence of low birthweights in the Indian subcontinent over the last 10,000 years, through both genetic and plastic mechanisms. BP, before present. This figure is available as part of a downloadable slideset

First, fetal growth is primarily determined by maternal lean mass and basal metabolic turnover, rather than adiposity, which funds lactation [42]. On this basis, height declines alongside thin physique would have increasingly constrained maternal nutritional investment during pregnancy. Second, the dimensions of the obstetric pelvis are correlated with maternal height [43]. Falls in adult height may therefore drive declines in pelvic size, thus impacting fetal growth, either through plastic responses or genetic co-adaptation (Fig. 4). However, note that other populations showing stable short stature since the Paleolithic era have relatively wide pelvic dimensions, suggesting compensatory adaptations [44].

There is some evidence for long-term adaptations of growth patterns in South Asia. Analyses of inter-ethnic unions indicate that paternal genotype contributes to the smaller size of Indian vs European neonates, and maternal genotype may contribute similar effects [45]. Similarly, average pelvic dimensions of South Asians remain small relative to those of other populations [46]. However, plastic responses may also be involved, as suggested by small secular increases in Indian birthweight over several decades that remain trivial relative to the long-term decline [47].

Long-term declines in maternal height may have been driven by several ecological stresses associated with the shift from foraging to sedentary agriculture, including population growth, food insecurity and an increased burden of infections [30]. A shift from meat- to grain-based diets higher in carbohydrate and lower in protein could have exacerbated these effects, as might the subsequent emergence of vegetarianism [30, 48].

The persisting thin physique is more difficult to explain. Possible adaptive explanations include heat stress, unpredictable food supply and regular monsoon-provoked famines [30]. It is notable that other populations exposed to regular fluctuations in food supply, including east African and Australian Aboriginal populations, show similar phenotypes (low lean mass and a predisposition to abdominal adiposity) and high diabetes susceptibility in obesogenic settings [10].

Likewise, higher birthweights in populations that live at high latitude may reflect positive selection on lean mass. According to thermodynamic theory, cold climates favour greater lean mass to promote heat production, whereas hot climates favour lower lean mass to promote heat loss. Climatic selection may act especially strongly in early life, when the ratio of surface area to mass is inherently high. Consistent with these predictions, both birthweight and adult lean mass are inversely associated with mean annual temperature across populations [49, 50], and high-latitude populations have both larger organs and greater muscle mass [30], each of which reduces diabetes susceptibility.

Regarding metabolic load, ethnic groups from contrasting geographical regions show variability in both whole-body adiposity and its anatomical distribution [51, 52]. Whole-body adiposity broadly is inversely associated with mean annual temperature, similar to lean mass [50]. However, adiposity also increases in association with mean annual precipitation (a marker of local food availability), and with inter-annual volatility in temperature (a marker of spikes in the burden of infections) [53].

The latter association is of particular interest. Ethnic variability in fat distribution is unlikely to have arisen through the selective pressure of famine or food insecurity as, although this stress varies geographically in its likelihood, its crude metabolic impact (weight loss) must be relatively uniform across all environments [51]. However, populations differ both in their local burden of infectious disease and in the optimal immune responses. Adipose tissue plays a key role in immune response, providing both energy and molecular precursors for immune agents. Since different infections target different anatomical regions of the body, adaptation of adipose tissue location and metabolism are expected [51]. For example, gut infections may favour immune responses in the viscera, whereas *Plasmodium* infection may favour storing lipid in muscle tissue, to provide the substrate for the adaptive response of fever [40].

According to the variable disease selection hypothesis, therefore, geographic differences in local disease burden may have shaped ethnic variability in the anatomical distribution of adipose tissue and its metabolic activity [51]. Ecogeographic analyses support the notions that pathogen burden is associated with abdominal adiposity and that fat distribution varies by geographical location [54]. This hypothesis may help explain why, for example, populations such as South Asians have an elevated susceptibility to visceral adiposity and also show greater increases in insulin resistance per kg of fat compared with Europeans [55].

In the reverse direction, diabetes may itself alter the susceptibility of humans to infections. For example, individuals with diabetes have an elevated risk of tuberculosis and severe dengue fever [56, 57], and a dose–response association of blood glucose levels with susceptibility to malaria suggests that greater substrate availability may fuel *Plasmodium* growth [56]. Conversely, people with diabetes have lower risk of helminth infections [57]. Although these associations remain poorly understood, especially in tropical environments where infectious diseases are most prevalent, the available data suggest that both circulating fuel levels and adipose tissue biology interact with immune function. These emerging data support the notion that ethnic variability in adiposity and metabolism has been shaped by the local burden of infectious diseases, thereby contributing to variability in the manifestation of diabesity.

### Life course plasticity in capacity and load

Just as the forms of phenotypic variability that emerge through genetic adaptation represent ‘solutions’ to ‘ecological problems’, life course plasticity in both metabolic capacity and load solves similar problems over shorter timescales.

However, it is crucial to remember that natural selection favours phenotypic traits that promote fitness, not health [38]. On this basis, successive metabolic adjustments through the life course that individually promote fitness may be cumulatively detrimental to health [20]. This helps to explain why diabetes risk shows strong associations with markers of malnutrition, poverty and inequality. As life ‘gets worse’, health is steadily sacrificed in order to promote survival and reproduction.

Regarding metabolic capacity, numerous stresses can reduce maternal nutritional investment in the fetus, with implications for later diabetes risk. Such stresses include overt famine, as demonstrated by follow-up studies of the Dutch Hunger Winter, Biafran conflict and the Great Chinese Famine [58–60]; seasonal fluctuations in food supply [61]; and disruption of maternal metabolism by infectious disease [2]. In modern environments, low position in the socio-economic hierarchy provides a composite marker of many individual stresses and is a strong correlate of low birthweight. On a similar theme, recent work from Malawi has shown that severe child malnutrition is associated with long-term deficits in metabolic capacity, but there is no direct impairment of glucose homeostasis provided that metabolic load also remains low [62].

Increasingly, those exposed to undernutrition in early life are later exposed to the obesogenic niche and develop high metabolic load, representing a ‘dual burden of malnutrition’ at the level of the individual. There is substantial evidence that obesity from childhood onwards is more ‘toxic’ among those previously undernourished compared with those with better early-life nutrition [63]. In adults, for example, short stature is a well-established risk marker for diabetes [30].

**Fig. 5:**
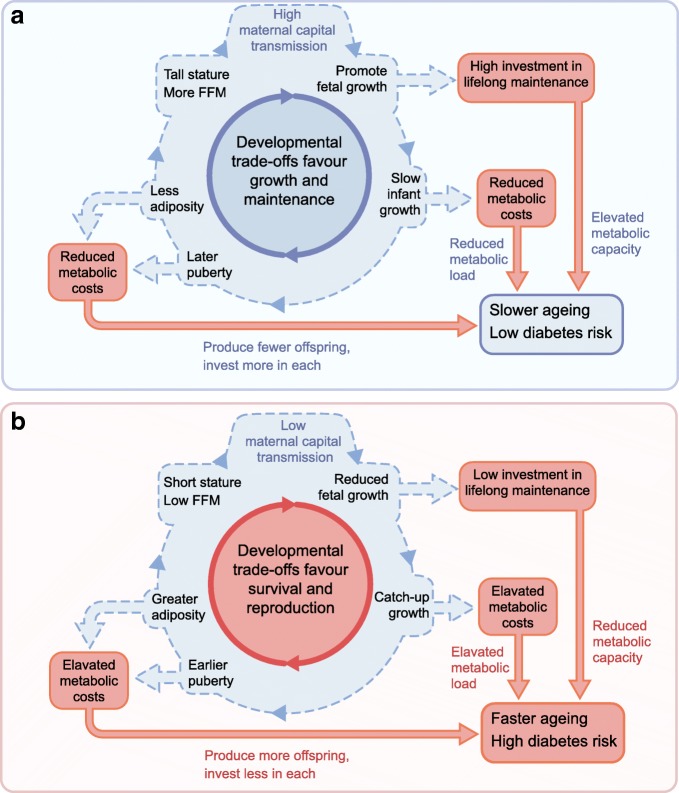
Schematic diagram of contrasting intergenerational cycles, characterised by developmental trade-offs that favour either (a) growth and maintenance or (b) survival and reproduction. Blue arrows represent life course developmental associations, red arrows represent cardiometabolic effects. These contrasting patterns favour different levels of maternal capital transfer to offspring, and favour the occurrence of similar trade-offs across generations. FFM, fat-free mass. This figure is available as part of a downloadable slideset

The ‘dual burden’ clearly increases diabetes risk, but the underlying metabolic responses can individually be considered as enhancing fitness in tough environments. The thrifty phenotype promotes early survival but at a cost of reducing life expectancy, which in turn reduces the ‘pay-off’ for investing in the long-term maintenance of health [20]. Early undernutrition therefore favours increased energy allocation to ‘defence’ and reproduction, to promote genetic replication before death occurs. This helps explain why, when those born small subsequently encounter energy-dense diets, they do not fully catch up in height and lean mass, but, rather, tend to experience accelerated maturation, associated with catch-up in weight, earlier puberty, higher levels of total-body and abdominal adiposity and an earlier emergence of diabetes [64]. This scenario gives rise to intergenerational patterns of risk transmission, with individuals diverging between two extremes (Fig. 5). High levels of maternal investment allow the offspring to invest in growth and maintenance, in order to reap fitness pay-offs in the longer-term future. Low levels of maternal investment drive the offspring to invest in defence and rapid maturation, which results in low birthweight being followed by elevated levels of body fat and central adiposity. These patterns were all demonstrated in a study of South Asian women living in the UK [64].

### The obesogenic setting

Given the responses described above, it is not difficult to understand why obesogenic environments are so strongly associated with diabetes. Despite major efforts, undernutrition in early life remains a severe health burden in most low- and middle-income countries, such that many individuals start life with low metabolic capacity. Through nutrition transition, this low capacity is increasingly exposed to multiple factors promoting rapid weight gain from childhood onwards, including sedentary behaviour and diets that challenge fuel homeostasis. Increases in maternal obesity are complicating and exacerbating these adverse effects. Given long-term selection on immune defence, it is unsurprising that a rapid secular shift from low-energy, high-pathogen environments to high-energy, low-pathogen environments results in large secular increases in abdominal fat, indicated by waist girth [54]. Indeed, efforts to reduce helminth infections can paradoxically increase insulin resistance [65], indicating the release of more fuel for the human host’s tissues.

Importantly, although most attention is placed on individuals and their lifestyles, much that drives diabetes risk lies beyond the control of the individual. I have used the concept of the ‘metabolic ghetto’ to elucidate how, at many levels, nutrition is actively used as a means to manipulate individuals and populations for economic benefit, through pressures that coerce unhealthy behaviour [10]. Here, health is traded off not against evolutionary fitness, but against profit. While the discussion above focused on biological components of diabetes risk, we must not ignore how powerful economic and political factors playing out over lengthy historical periods have also shaped diabetes susceptibility within and across populations, which then interact with rapid environmental changes resulting from transformation of food systems, urbanisation and other aspects of economic development. In this sense, we are observing recent and contemporary components of niche construction, through which humans themselves are shaping the selective pressures acting on human metabolism [66].

### Attenuating the diabesity pandemic

Beyond helping us to understand variability in diabetes risk, what can an evolutionary perspective contribute to efforts to tackle the diabesity pandemic?

First, within any individual generation, there is a limited direct opportunity to promote metabolic capacity. Supplementary feeding programmes during pregnancy can reduce the prevalence of low birthweight but produce relatively small increases in average birthweight [67, 68]. Such interventions may even promote maternal fertility, at a cost to child nutritional status [69]. Overall, secular trends in birthweight tend to be modest, suggesting that several generations would be required to see major change [10, 70]. In the short term, higher birthweights may contribute to another global pandemic, that of Caesarean sections [71, 72].

A more promising indirect opportunity to promote metabolic capacity is to delay the age of women’s reproduction, which in many populations requires delaying age at marriage [73]. Adolescent mothers have an elevated risk of delivering small neonates [74], reflecting both low BMI and their incomplete pelvic growth. Another important avenue is to reduce rates of malnutrition (wasting and stunting) in postnatal life, when the pancreas and muscle mass are still developing. In this context, interventions could target seasonal spikes in food insecurity and infection risk to decrease the risk of infant malnutrition.

However, efforts to tackle global diabesity are likely to have little success if they do not reduce the metabolic load associated with obesity and unhealthy lifestyles. This is particularly urgent given the intergenerational associations described above. The severity of the diabesity epidemic requires fundamental action: the entire human food system needs to be redesigned to address a constellation of problems, including rural poverty, food insecurity and unhealthy diets. Similar approaches must target sedentary and stressful lifestyles. The contemporary diabesity epidemic indicates how we have so far failed in this effort.

## Electronic supplementary material

ESM (PPTX 710 kb)

## Abbreviation

GDM: Gestational diabetes mellitus
