# Supplementary material for: The diabesity epidemic in the light of evolution: insights from the capacity–load model
Source: Diabetologia. 2019 Aug 27;62(10):1740–50. doi: 10.1007/s00125-019-4944-8 (PMC6731192; doi:10.1007/s00125-019-4944-8)
Supplement: Supplementary file 1 — (PPTX 710 kb) [file 125_2019_4944_MOESM1_ESM.pptx]

## Slide 1
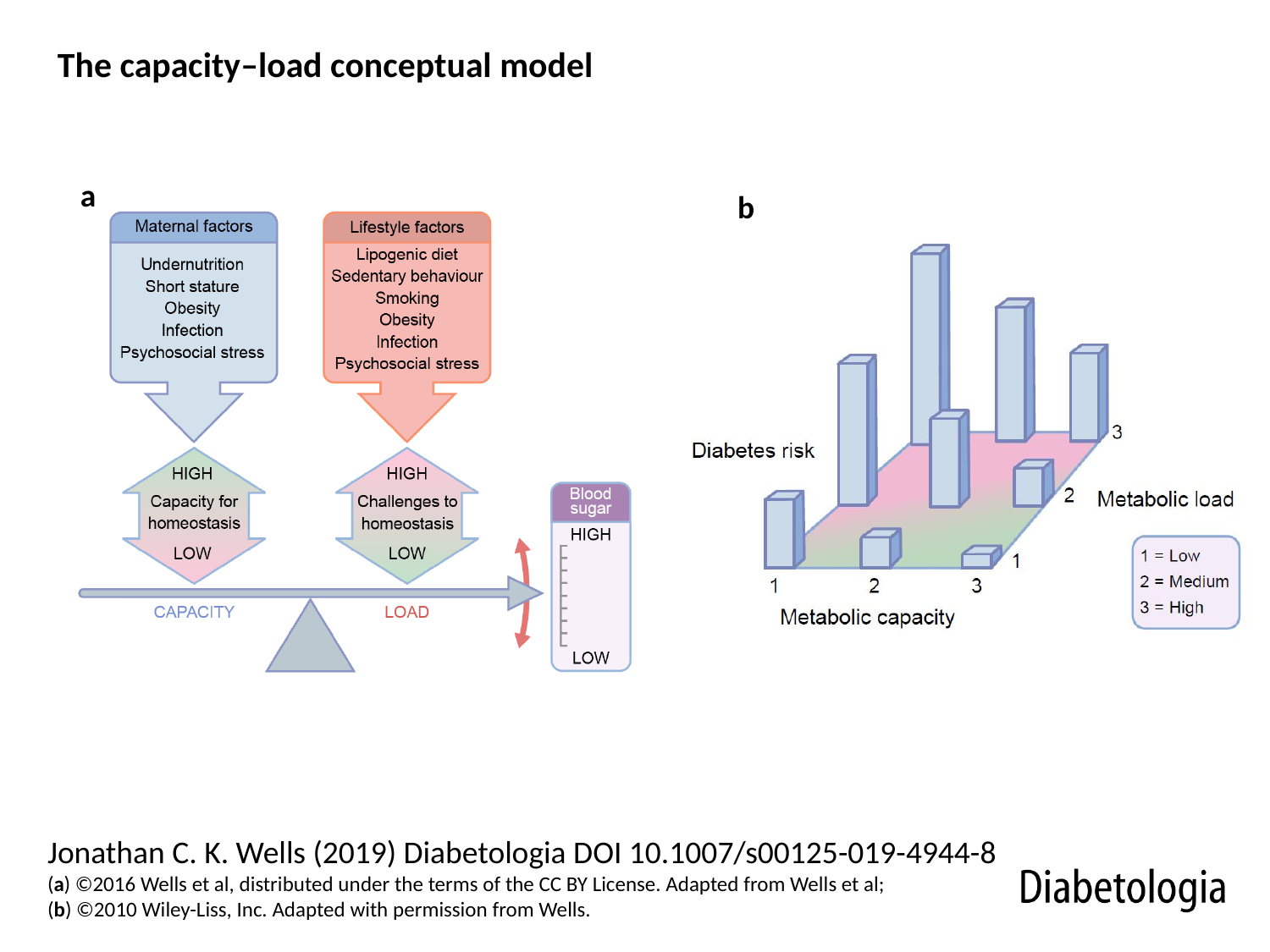

The capacity–load conceptual model
a
b
Jonathan C. K. Wells (2019) Diabetologia DOI 10.1007/s00125-019-4944-8
(a) ©2016 Wells et al, distributed under the terms of the CC BY License. Adapted from Wells et al;
(b) ©2010 Wiley-Liss, Inc. Adapted with permission from Wells.

## Slide 2
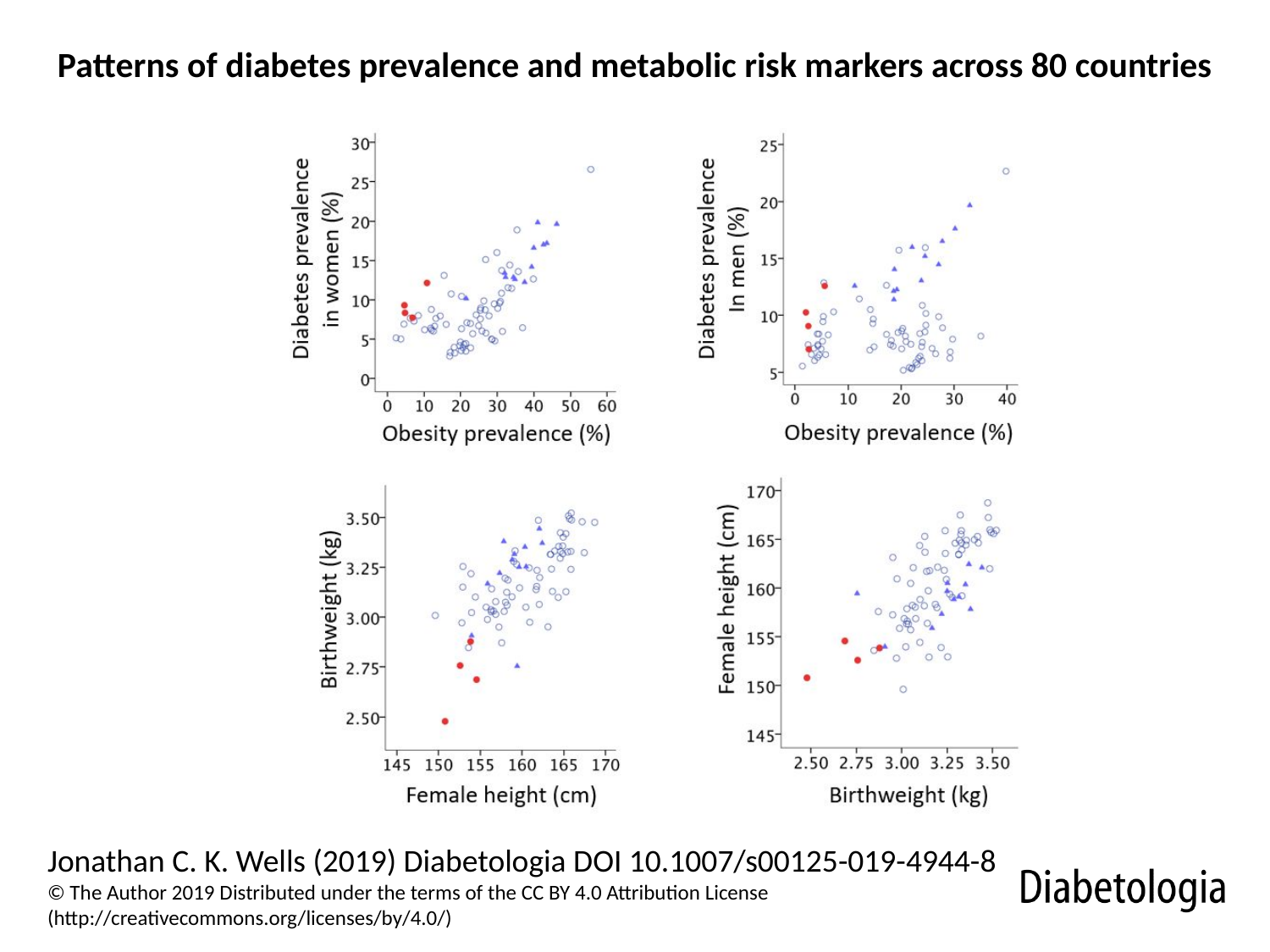

Patterns of diabetes prevalence and metabolic risk markers across 80 countries
Jonathan C. K. Wells (2019) Diabetologia DOI 10.1007/s00125-019-4944-8
© The Author 2019 Distributed under the terms of the CC BY 4.0 Attribution License (http://creativecommons.org/licenses/by/4.0/)

## Slide 3
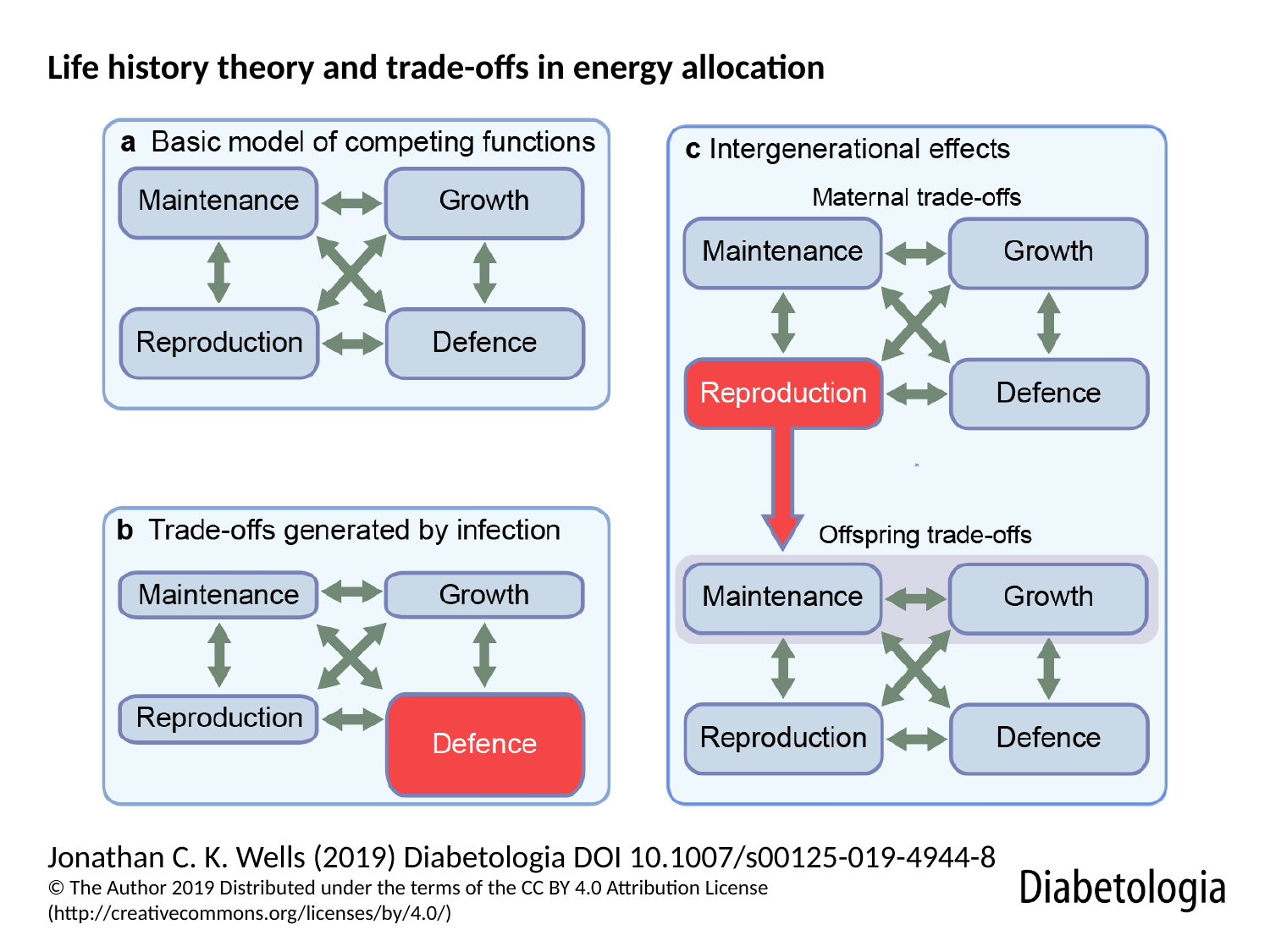

Life history theory and trade-offs in energy allocation
Jonathan C. K. Wells (2019) Diabetologia DOI 10.1007/s00125-019-4944-8
© The Author 2019 Distributed under the terms of the CC BY 4.0 Attribution License (http://creativecommons.org/licenses/by/4.0/)

## Slide 4
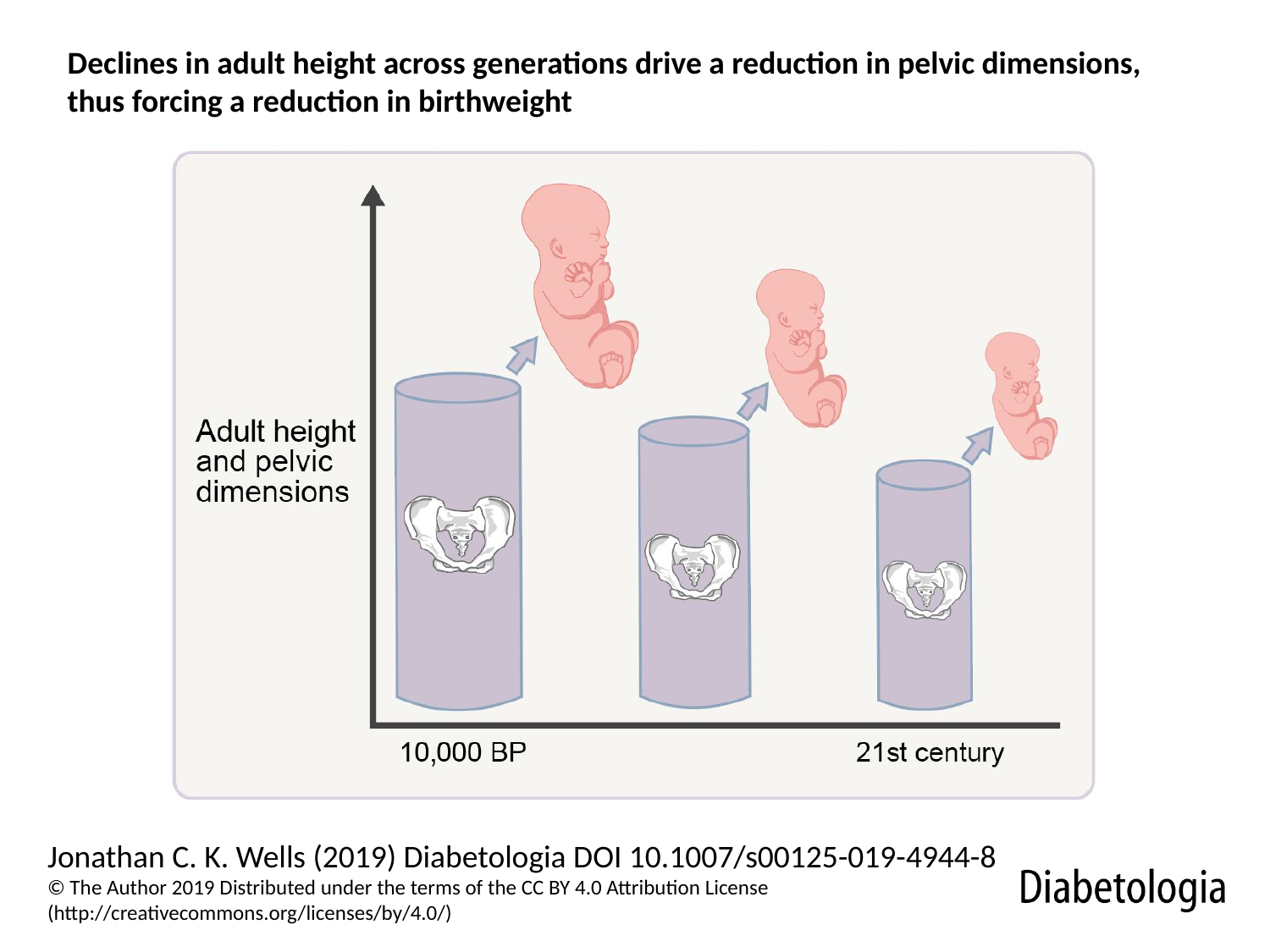

Declines in adult height across generations drive a reduction in pelvic dimensions, thus forcing a reduction in birthweight
Jonathan C. K. Wells (2019) Diabetologia DOI 10.1007/s00125-019-4944-8
© The Author 2019 Distributed under the terms of the CC BY 4.0 Attribution License (http://creativecommons.org/licenses/by/4.0/)

## Slide 5
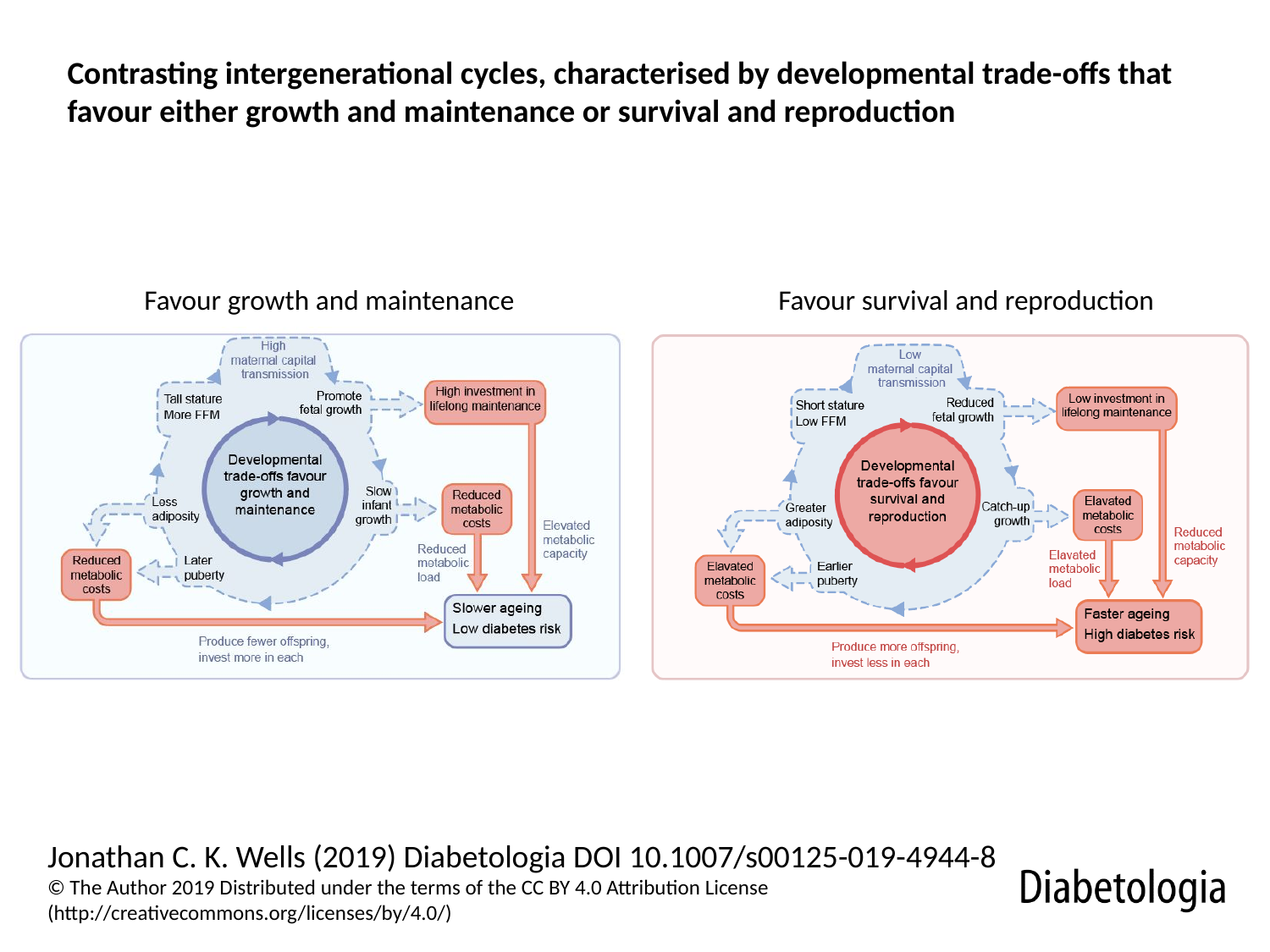

Contrasting intergenerational cycles, characterised by developmental trade-offs that favour either growth and maintenance or survival and reproduction
Favour growth and maintenance
Favour survival and reproduction
Jonathan C. K. Wells (2019) Diabetologia DOI 10.1007/s00125-019-4944-8
© The Author 2019 Distributed under the terms of the CC BY 4.0 Attribution License (http://creativecommons.org/licenses/by/4.0/)
